# Reducing Barriers to Language Assistance During a Pandemic

**DOI:** 10.1007/s10903-021-01251-2

**Published:** 2021-07-30

**Authors:** Erin Mulpur, Travis Turner

**Affiliations:** Houston Methodist Global Health Care Services, Houston Methodist Hospital System, 6560 Fannin Street, Suite 570, Houston, TX 77030 USA

**Keywords:** Language assistance, Interpretation, Language rights, Coronavirus, Limited english proficiency, COVID-19

## Abstract

This “Notes from the Field” article discusses language assistance within healthcare during the COVID-19 public health crisis. Providing adequate language assistance within healthcare is fundamental. At Houston Methodist we learned that we could leverage existing technologies to address language needs of our COVID-19 patients with limited English proficiency during the pandemic when personal protective equipment was in limited supply across the United States. By leveraging the use of our existing technologies (ex. Telephone interpretation with wearable communication devices) we increased utilization of language assistance for our patients with limited English proficiency. We urge other healthcare organizations to re-evaluate their language assistance programs and leverage similar technologies to empower both clinicians and patients.

## Notes From the Field

The novel Coronavirus has shaken the walls of our health care infrastructure in the United States as unprecedented economic, social, and political factors continue to negatively impact the health of our nation. As large health care organizations continue to be slammed by the crashing waves of the pandemic, it can be easy to lose focus on disadvantaged populations in the wake of it all. However, it is essential to continue caring for a diverse population of patients effectively and equitably, including patients with limited English proficiency (LEP).

Patients with LEP experience difficulty understanding the care they are receiving and can therefore struggle to advocate for themselves, understand their treatment options, or establish expectations. Low-income and disadvantaged persons often suffer disproportionately during natural disasters and epidemics [1, 2], and the health disparities experienced by those with LEP will only increase in frequency if our current approach to health information delivery does not adapt accordingly [3]. Providing adequate language assistance is particularly important in large, multi-cultural cities. Houston is the fastest growing multi-cultural city in the nation with the largest medical center in the world [4]. The Texas Medical Center also draws in medical tourism, with patients traveling from all around the world to receive care. Recent reports show that in Houston, just 54% of persons 5 years and older speak only English at home, compared to 79% in the United States [4]. Under Title VI of the Civil Rights Act of 1964, Medicaid and other federally funded programs must provide assistance to persons with LEP [5]. However, less than 70% of all US hospitals offer language-concordant care [6] and fewer than one-third of outpatient physicians report regularly using a trained professional interpreter [7]. Ultimately, language rights are human rights and our LEP community needs us to band together and take action.

Spanish is the second most commonly spoken language in the United States [8] and the predominant language spoken by the patients at Houston Methodist Hospital who need language assistance. Spanish-speaking Hispanics were at greatest risk of exposure to the H1N1 flu during the 2009 epidemic. These patients also had less access to health care, and these disparities remained significant even after controlling for other demographic and socioeconomic factors [9], which could be due in part to the language barriers they face.

Traditionally, language assistance at Houston Methodist has been provided by an in-person interpreter, which requires a scheduled appointment with multiple parties involved. Through advancements in technology over the years, we added telephone and video remote interpretation services to allow clinical staff to connect with patients on demand when we did not have an in-person interpreter available. However, the COVID-19 crisis has forced us to rethink how to make sure our patients understood the care they were receiving while protecting our staff from exposure. At the beginning of the pandemic, a critical focus for health care organizations was conserving personal protective equipment (PPE), which was in short supply. This required reevaluating the use of PPE for in-person interpretation as well as the problems posed by video interpretation devices. Clinical staff felt video devices were hard to use when in full PPE and added to an already crowded room, and they also required staff to sanitize them between patients. To help staff more easily communicate with their patients, two technologies were merged and leveraged.

Many hospital systems utilize wearable technologies for communication regarding patient care. Houston Methodist uses the Vocera Smartbadge communication device (Vocera Communications, Inc., San Jose, California, US), which is a secure, HIPPA compliant, hands-free, wearable badge. Vocera badges were already being utilized across the hospital system for communication between nurses, doctors, and other clinical staff, who found it very helpful for use while working with patients. Therefore, we embedded the telephone interpreting number into the Vocera badge phone directory to be used across our system. This allowed all practitioners to quickly, and without contaminating a device, call for language assistance when assisting a patient with LEP. It became as easy as saying, “Vocera, call Language Assistance.”

When we think of hand washing in health care, there are hand sanitizers every 5 feet in a hospital. Language assistance can be just as accessible. By merging telephone interpretation and wearable communication technologies, which are already walking around the campus on the chests of nurses and doctors, the same can be true of language assistance.

We were lucky to have put this in place prior to the second surge of COVID-19 patients in Houston. As reported by Vahidy et al., the number of Hispanic and Latino COVID-19 patients in the Texas Medical Center surged disproportionately starting around late May [10]. The wave of language assistance needed during this time was unprecedented, and we couldn’t have handled the volume without this vital integration. Due to this increased need and the convenience of language assistance being at one’s chest, the number of conversations utilizing telephone interpretation has increased tremendously. From the first quarter of 2020 to the second quarter, our average telephone interpretation minutes used per month more than doubled from 12,781 to 25,018. In the month of July alone, more than 46,000 min were used, with the top language needed being Spanish. Convenience has played a large role in the success of this strategy. Strategically embedding language assistance into something that is already integral to the workflow of our clinical staff has allowed for quick adoption and a high level of comfort with the technology. It was also noted that during this pandemic that re-education on the forms of language assistance has been vital to increase its utilization.

Practitioners have also commented that they feel a higher level of connection with the patient while using this form of interpretation. Having the device on the practitioner has placed the interpreter’s voice on their chest. The voice is coming from the practitioner; from their heart. This has added a layer of compassion and warmth to the tense situation of being under care in a hospital and not speaking the common language, often without a family member at the bedside due to the public health crisis. There is no more barrier, phone cord, tablet, glass door, or lack of understanding present at the bedside.

Despite its simplicity and convenience, we do recognize there are times at which this technology would not be appropriate. For example, if our patients with LEP need end of life conversations and a family meeting is required, utilizing in-person interpretation is extremely encouraged and beneficial. However, we also recognize that many healthcare organizations do not even offer any form of language assistance [6], so leveraging technology would be the easiest way for them to become compliant. This technology, like other remote interpreting options, protects interpreters from exposure to COVID-19 and other diseases; however, widespread adoption could reduce the use and, eventually, the availability of in-person interpreting services. In addition, more research must be done to determine the patients’ perspective on the use of this potential new model of care, especially in the post COVID-19 era. Conducting surveys and interviews of patients and family members after they receive language services could provide information on which forms of interpretation patients prefer and identify limitations and areas for improvement. Ultimately, providing language assistance to a patient should not be a burden, and the easier you make it, the better the outcome for both clinician and patient.

We can’t reiterate it enough; language rights are human rights. This was federally recognized with the Civil Rights Act in 1964, reiterated with the Americans with Disabilities Act in 1990, and again reinforced with the Affordable Care Act in 2010. Providing language assistance to our patients with LEP is essential to allow them to feel like active members of their care journey. However, we also understand adding more complicated layers to an already burdened clinician is not the answer either, and healthcare organizations must continually consider how we can ensure language assistance technology isn’t a burden to the patient or clinician. The margin for hospitals is minimal and our population of patients is becoming more and more diverse, requiring us all to consider what internal infrastructure can be leveraged to allow for cost effectiveness and ease of access to language assistance. By integrating our Vocera devices with our telephone interpretation, we have been able to better support our clinical staff and connect with our patients with LEP in ways that keep everyone safe. There are simple solutions that healthcare systems can consider in order to provide language assistance within their organization, but we must make this a priority for all. The time to act is now. We have the ability to influence change for this patient population that will have long-term positive effects on our population’s health now and in the future.
